# Estimating Occupational Exposure to VOCs, SVOCs, Particles and Participant Survey Reported Symptoms in Central Thailand Rice Farmers Using Multiple Sampling Techniques

**DOI:** 10.3390/ijerph18179288

**Published:** 2021-09-02

**Authors:** Saowanee Norkaew, Wantanee Phanprasit, Mark Gregory Robson, Susan Woskie, Brian T. Buckley

**Affiliations:** 1 Faculty of Public Health, Thammasat University, Khlong Nueng 12121, Thailand; 2 Department of Occupational Health and Safety, Faculty of Public Health, Mahidol University, Bangkok 10400, Thailand; wantanee.pha@mahidol.ac.th; 3 Department of Plant Biology, School of Environmental and Biological Sciences, Rutgers University, New Brunswick, NJ 08901, USA; robson@sebs.rutgers.edu; 4 Department of Public Health, Zuckerberg College of Health Sciences, University of Massachusetts Lowell, Lowell, MA 01854, USA; susan_woskie@uml.edu; 5 Environmental and Occupational Health Sciences Institute, Rutgers University, Rutgers, NJ 08854, USA; bbuckley@eohsi.rutgers.edu

**Keywords:** rice farmer, passive air sampling, hazard exposure

## Abstract

Thailand is known for its agricultural productivity and rice exportation. Most farms use small machines and manual labor, creating potential exposure to multiple health hazards. A cross-sectional study was conducted to measure pollutants liberated during preparation, pesticide application, and harvesting. Thirty rice farmers, mostly males from 41 to 50 years old, participated. The participant survey data showed that 53.3% of the respondents spent >2 h per crop on preparation, <1 h on pesticide application, and about 1–2 h harvesting; 86.7% of the respondents maintained and stored mechanical applicators at home, suggesting possible after-work exposures. Gloves, fabric masks, boots, and hats were worn during all activities, and >90% wore long sleeved shirts and pants. VOCs and SVOCs were collected using charcoal tubes and solid phase micro sample extraction (SPME). An analysis of the charcoal and SPME samplers found that 30 compounds were detected overall and that 10 were in both the charcoal tubes and SPME samplers. The chemicals most often detected were 1, 1, 1 Trichloro ethane and xylene. Additionally, farmers experienced the highest exposure to particulates during harvesting. These results demonstrated that farmers experience multiple exposures while farming and that risk communication with education or training programs may mitigate exposure.

## 1. Introduction

Thailand is known as an agricultural country and a major exporter of rice. Fertilizers, herbicides, and insecticides are used to try to help guarantee production quality, but most of the farms cultivated for rice production are medium to small in size and farming activities are generally conducted using small machines and manual labor. Farming activities may expose farmers to a variety of health hazards including dust, aerosols, and engine exhaust generated as part of normal farming practices. Previous studies showed that agricultural operations increase particulate concentrations in the atmosphere [1,2,3,4,5,6]. The California Air Resources Board also demonstrated that agricultural operations modify the atmospheric particulate matter (PM) concentration in California by about 25%. This estimate is based on measurements for a variety of operations (such as land planting, disking, and floating) [7]. Exposure from the mechanized farming equipment sampled in this study included tractors for land preparation, mechanical backpack sprayers for pesticide application, and harvesters for harvesting crops. The chemical contaminants emitted from gasoline (and diesel) engine farming equipment are volatile/semi-volatile organic compounds (VOCs/SVOCs), nitrogen compounds (e.g., Indole and Carbazole), metals, black carbon (BC), and PM that create an exposure risk [8,9,10]. The toxicity of both regulated and unregulated combustion engine pollutants may lead to various health effects. Benzene, formaldehyde, benzo(a) pyrene, and black carbon are classified as carcinogens by the International Agency for Research on Cancer (IARC). Several VOCs emitted during farming operations may act as precursors to the formation of secondary organic particles by atmospheric photo-oxidation [11]. In addition, epidemiological and environmental studies demonstrate that aerosol particles can contribute to a variety of human health problems. According to the World Health Organization (WHO), particle pollution contributes to approximately 7 million premature deaths each year, making it one of the leading causes of mortality worldwide [12]. Serious respiratory disorders—cardiovascular, respiratory, and allergic diseases including asthma, chronic obstructive pulmonary disease, pneumonia, and possibly tuberculosis; lung cancer risk; and nervous system disorders have all been linked to aerosol exposure [13,14,15,16,17]. The consequences of omnipresent indoor–outdoor aerosol exposure depend on the type of aerosol present, the type of outdoor environment, the duration of time spent exposed to the aerosol, age, gender, susceptibility, and many other factors [18,19,20,21]. Understanding aerosol concentrations emitted into the atmosphere from agricultural activity is important [22] because, in some areas, it may be due to agricultural activities, but limited data are available about the chemical contributions to this agricultural aerosol [23]. A large fraction of PM2.5–PM10 can be generated from mechanical processes: windblown dust, dust from rural roads and from activities such as harvesting, and disking [24]. The size and mass distribution of particles may be defined by emissions from various sources, including agricultural burning and ambient sources [23].

The objective of this study was to explore the air quality in an agricultural area including VOCs, SVOCs, and particulate concentrations during farming activities and to identify the symptoms related to pollutants [13,14,15,16,17] among rice farmers.

## 2. Materials and Methods

### 2.1. Study Site

**Figure 1:**
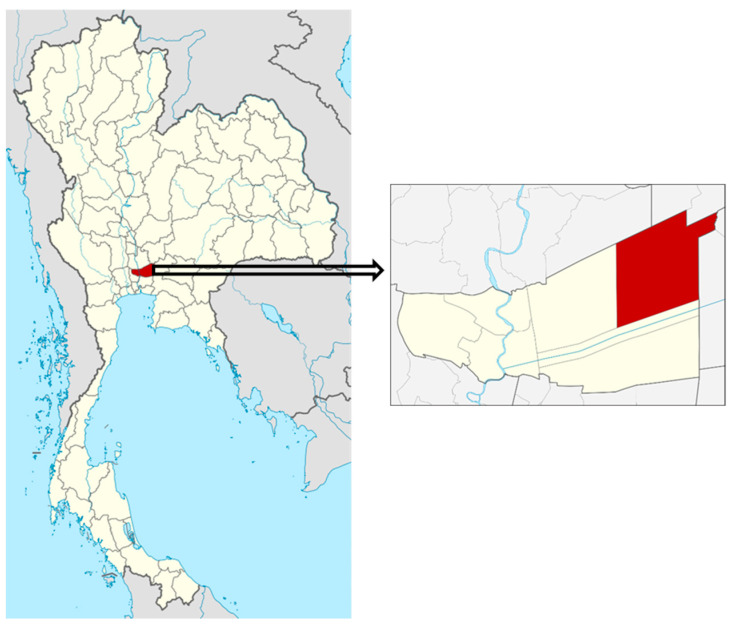
Study site [27,28].

The study was carried out in Nong Suea District, an area in Pathumthani Province, Thailand, where the farmer population is dense [25], as shown in Figure 1. From the Agricultural database year 2016/2017, it was reported that 2450 rice farmers had 26,300,000 Rais or 10,395,256.917 acres [25,26]. Normally, farmers in this area farm rice year-round, with a rice crop round taking 3–4 months. Immediately after rice is harvested, the farmers continue with the cycle of planting a new crop in the same area. Rice farmers use their own farm machines or rent machines from others. This study was focused only on farmers who used gasoline-fueled equipment in all processes of rice farming.

### 2.2. Study Design

A cross-sectional study was conducted to investigate the relationship of the farmers’ exposure to airborne VOCs, SVOCs, and particulate matter as well as the related symptoms during one cycle per farm of rice farming activities between October 2018 and August 2019. A questionnaire was used to collect data (e.g., the farmers’ general information, and VOCs and particulate matter related symptoms), while air samples were collected to estimate their exposure to air pollutants. The correlations between the concentration of all chemical contaminants, farming activity risk factors, and health status were determined by statistical analyses including descriptive statistics, Pearson correlations, and the Mann–Whitney U test.

### 2.3. Participants

The study population was rice farmers living in Nong Suea District, Pathumthani province. Sample size calculation was based on the main objective of the study to identify the types of chemical exposure among rice farmers. The inclusion process for study participants was divided into two parts: screening by questionnaire and then measurement of the chemical exposure. A multi-stage area sampling technique was used for the participants selected. An invitation letter was sent to Nong Suea District Administrative Organization and Sub District Health Promoting Hospital for research participants: farmers who are interested could apply, and research participants were selected by the criteria. Of the 246 farmers who completed the questionnaires, 30 farmers were invited to participate in the airborne VOCs, SVOCs, and particulate exposure measurements. A farmer was invited if they used gasoline engine equipment in all processes, more than 18 years, residents of the study area for more than 1 year, and willing to participate in this study.

### 2.4. Materials and Methods

#### 2.4.1. Questionnaire

The questionnaire developed by the researcher and adopted from Laden F. et al. and Issa Y. et al. previously employed in other studies [13,29] was used. The questionnaire had three parts: (1) farmers’ general information; (2) their work practices (i.e., farming tasks and work duration), operating procedures (i.e., mechanical applications usage and storage), and personal protective equipment (PPE); and (3) any symptoms that may be related to VOCs, SVOCs, and particle exposure, i.e., symptoms of the skin, respiratory system, eyes, and neuromuscular and central nervous systems. The questionnaires were administered in person, and each participant was interviewed by a well-trained personnel on the community meeting.

#### 2.4.2. Air Sampling Instruments

VOCs sampling and analysis: two kinds of samplers were used.

**Figure 2:**
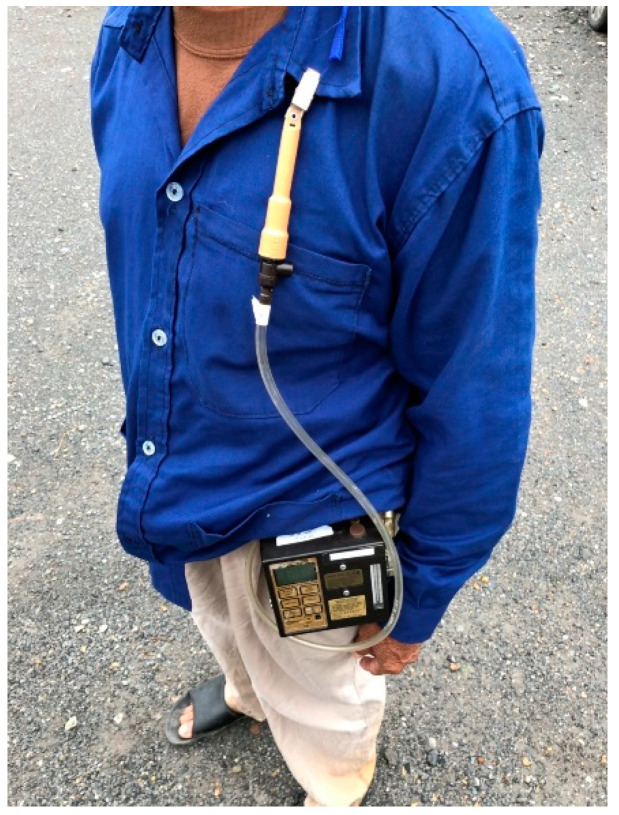
Absorbent tube sampling application.

**Figure 3:**
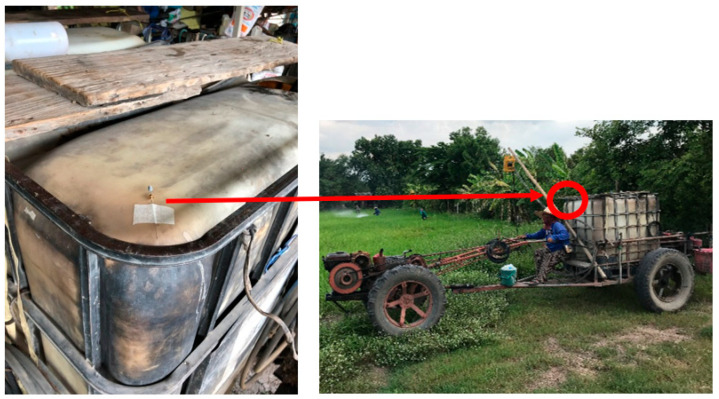
SPME sampling application.

1. Absorbent tube. NIOSH method #1501 was employed for VOC sampling and analysis [30]. Each personal sampling pump—SKC 224-PCXR8 with a representative sampler, 100 mg/50 mg coconut shell charcoal tube, in line was calibrated to obtain the flow rate of 0.2 L/min. The sampler equipment was attached to the farmer’s clothing in their breathing zone as shown in Figure 2. The air sampling was run while the farmer worked on their tasks, recalibration was conducted immediately after the air sampling ended, and the average flow rate before and after air collection was used for concentration calculation. The samples were capped and packed for shipment to the laboratory, where they were stored in the refrigerator at 4 °C until analysis within 30 days of sampling.
2. Solid-Phase Micro-Extraction (SPME). The SPME, a passive but quick and universal sampling technique, does not require a pump or the use of organic solvents for analyte extraction and is used for the determination of various classes of pesticides and other VOCs and SVOCs in aqueous media or in other samples [31]. It is sensitive and convenient for field or laboratory use since equilibrium is quickly attained by adjusting factors including temperature, fiber type and exposure time, volume of sample, salt concentration, pH, and agitation [32]. For this study a 50/30 µm Divinylbenzene/Carboxen/Polydimethylsiloxane (DVB/CAR/PDMS), StableFlex/SS (2 cm), Manual Holder, gray fiber (SUPELCO, PA) fiber was used. The Solid-Phase Micro-Extraction (SPME) samples were placed beside farmers when in a sitting position while working and was exposed to the air beside the farmer’s working area, as shown in Figure 3. At the completion of sampling, the SPME fiber was retracted into the needle, putting the top of the fiber and the tip of the needle at the same position. The SPME fiber was stored in a glass tube with plastic caps and packed for shipment. All samples were then transported from the field by the researchers.

**Figure 4:**
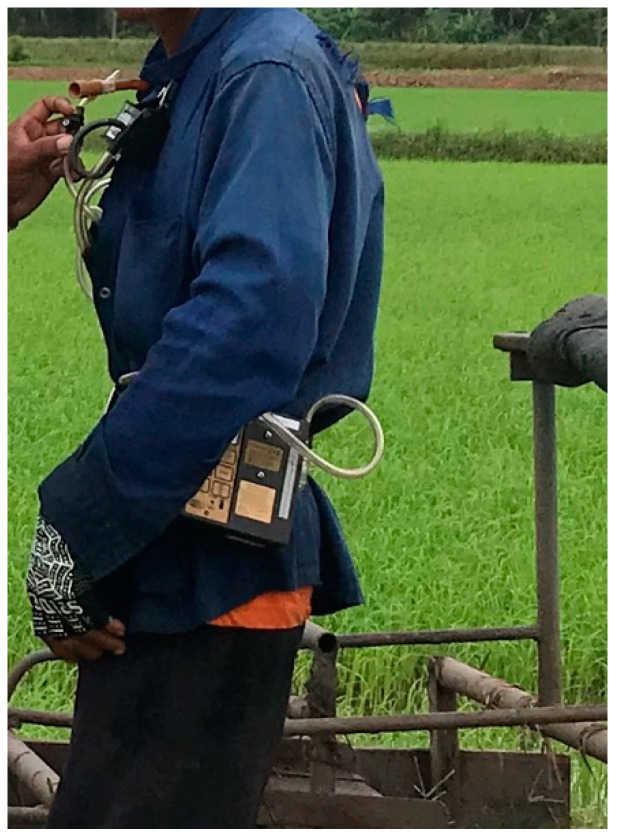
Particulate matter sampling application.

Particulate matter: NIOSH method #0600 was employed using a 37 mm PVC filter with an aluminum cyclone. The sampler was connected to a personal pump—SKC 224-PCXR8—and calibrated to obtain a flow rate of 2.5 L/min. The equipment was attached to the farmer’s clothing near their breathing zone, as shown in Figure 4. The air sampling was run while the farmer worked on their tasks and was recalibrated after the air collection finished. All filter samples and blanks were weighed before and after sampling on an analytical microbalance (Mettler Toledo: MX5) with a sensitivity of 0.001 mg.

Air sampling was conducted by a researcher in concert with trained assistants during each activity: land preparation, pesticide application and harvesting. All three air sampling instruments (the absorbent tube, SPME, and a filter) were collected from each participant. All 90 samples were supposed to be collected, including 30 absorbent tubes, 30 SPME fibers, and 30 filters, one for each type of sampling from each farmer. Unfortunately, many of the SPME fibers broke after deployment so only 5 SPME samples were analyzed although the other 60 samples (30 sorbent tubes and 30 filters) were analyzed.

#### 2.4.3. Air Sample Analyses

**Figure 5:**
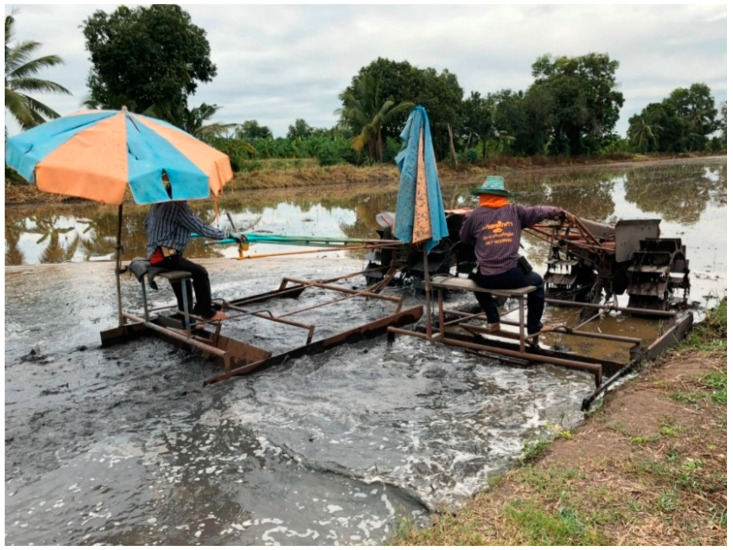
Land preparation.

**Figure 6:**
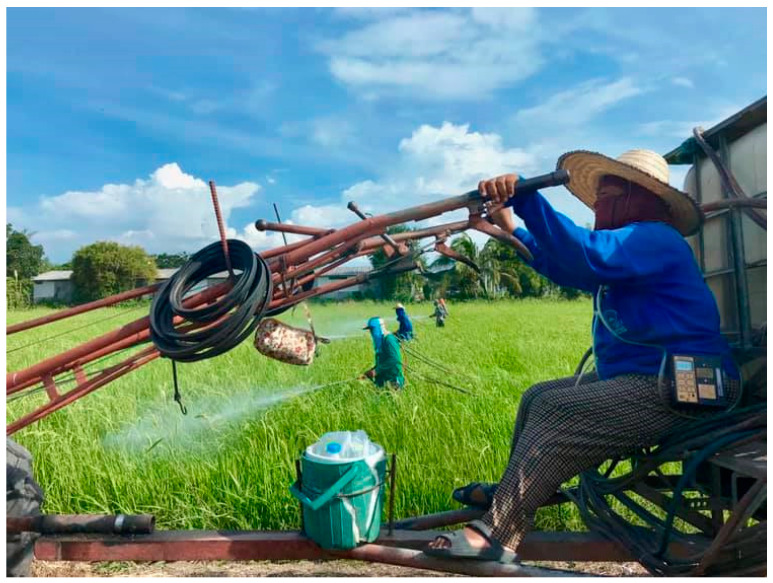
Pesticide spraying.

**Figure 7:**
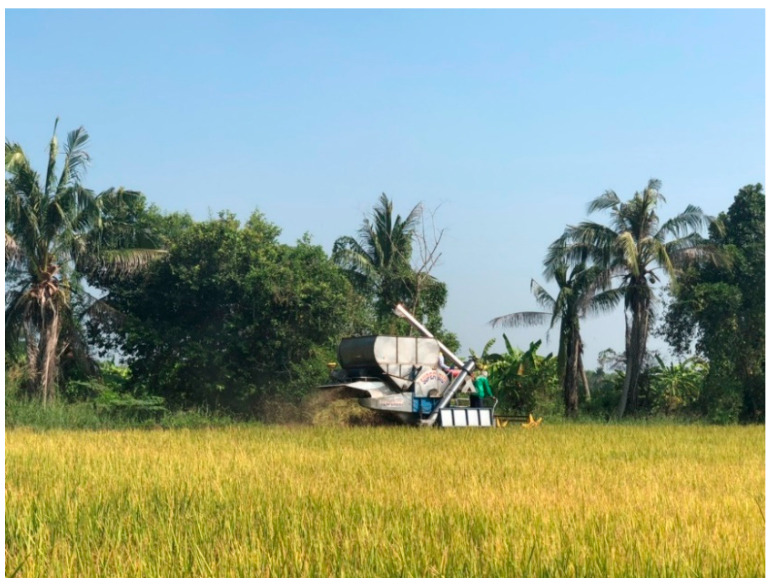
Harvesting.

All VOC air samples (solid charcoal sorbent tube and SPME) were shipped to a chemical laboratory at Thammasat University for analysis. For the solid charcoal sorbent tubes, the samples were analyzed by gas chromatography with a flame ionization detector (Clarus 600 T GC/FID; Perkin Elmer, Santa Clara, CA, USA), while the SPME samples were analyzed by gas chromatography with mass spectrometer (Clarus 600 T GC/MS; Perkin Elmer, USA) equipped with a J&W DB-VRX 20 m microbore capillary column with a 0.18 μm thick film (Agilent Technologies, Santa Clara, CA, USA). In all 49 VOCs, the components were analyzed against an EPA 502/524 volatile organic calibration mix. The VOCs included those with known health effects: Trichloromethane, Benzene, Trichloroethylene, Toluene, Ethylbenzene, Xylene, and Styrene. For respirable dust, each filter was weighed, including the field blanks, using a digital balance The sampling collection activities are shown in Figure 5, Figure 6 and Figure 7.

## 3. Results

The general profile and sociodemographic characteristics of the participants are presented in Table 1. All of the participants were male; the majority (33.3%) of them were aged 41–50 years old, and their highest level of educational attained was primary school (56.7%). Most of the participants had an income of less than 100,000 Baht per year (3200 USD), and more than half (56.7%) were currently smoking. About 43.3% of the respondents had congenital or underlying diseases, and 30.0% of them were currently on medication.

The farming process and work characteristics of the participants are presented in Table 2. More than 50% of the respondents occupied less than 10 rais (a Acres). Most of the respondents took more than 2 h for land preparation, less than 1 h per application for pesticide spraying, and 1–2 h for harvesting. About 36.7% of the respondents had less than 10 years of growing experience and in the use of mechanical applicators. The participants reported that they used several types of agrochemicals for a crop, for example, glyphosate, chlorpyrifos, profenofos, and carbosulfan.

Ninety percent said they always read the instructions and checked the equipment prior to use. Approximately, 86.7% of respondents prepared and stored mechanical applicators at home. More than 90.0% of respondents cleaned spraying equipment after work, and most of them never smoked nor drank alcohol while working. They wore gloves, masks, boots, and hats during any farming activity, and more than 90% of them always wore long-sleeved shirt and pants. The respondents reported that they used fabric/leather gloves and fabric masks while spraying. None used chemically resistant or commercial masks.

Personal air samples were collected from each participant to be analyzed for VOCs and SVOCs. Due to the fragility of the SPMEs, many fiber needles were broken during the sampling. Personal samples using the charcoal sorbent tube displayed a ruggedness of this sampling technique, which the SPME sampler did not enjoy. A previous study demonstrated that charcoal tube sampling might be comparable to SPMEs for quantitative assessment of exposure under the right conditions [33]. Fibers were deployed for all thirty farmers. Only five SPME samples were completed: one during land preparation, three after pesticide spraying, and one after harvesting. Overall, thirty chemicals were detected between the two techniques, with ten were found using both techniques. However, not all chemicals were found in all samples. These components were (1) 1, 2 Dichloroethaene; (2) Methlylene Chloride; (3) Trichloromethane; (4) 1, 1, 1 Trichloroethane; (5) Benzene; (6) 1, 2 Dichloro Propane; (7) Toluene; (8) 1, 3 Dichloro Propane; (9) Dibromo Chloro Methane; (10) 1, 2 Dibromoethane; (11) Ethylbenzene; (12) o-Xylene; (13) m-Xylene; (14) p-Xylene; (15) Styrene; (16) 1-Methyl Ethyl Benzene; (17) Propyl Benzene; (18) 1-Chloro-3-Methyl-Benzene; (19) 1-Chloro-2-Methyl-Benzene; (20) 1-Ethyl-3-Methyl Benzene; (21) Tert-Butyl Benzene; (22) 1, 3, 5-Trimethyl Benzene; (23) 1-Methyl Propyle Benzene; (24) 1, 3-Dichloro Benzene; (25) 1, 4-Dichloro Benzene; (26) Butyl Benzene; (27) 1, 2 Dibromo-3-Chloro Propane; (28) 1, 3, 5 Trichloro Benzene; (29) Naphthalene; and (30) 1, 2, 3 Trichloro Benzene. However, Methlylene Chloride; Ethylbenzene; o-Xylene; m-Xylene; p-Xylene; Stylene; 1-Methyl Ethyl Benzene; Tert-Butyl Benzene; 1, 3, 5-Trimethyl Benzene; 1-Methyl Propyle Benzene; and 1, 4-Dichloro Benzene were found both in a sorbent tube and in the SPME samples. Table 3 presents only the chemicals detected in charcoal tubes, and the chemical concentrations in the percentage of occupational exposure limits (OEL) if there is one.

The quantification of SPMEs in the field samples was not performed because of the required assumptions on how much air came in contact with the needle. The analytes absorbed on the needle can be quantified, and quantitative measurements are frequently performed on a water sample [40,41]. Some investigators have included air pumps to fix the amount of air that the needle’s sorbent material contacts [42]. While absolute quantitation of VOCs and SVOCs in passive air samples is difficult at best, the relative concentration measurement of these analytes in the air is certainly possible. For example, if a needle is deployed for 8 h on day one and then another needle is deployed for 8 h in the same location on day two, it is reasonable to assume that, if the peak area on a GC or GC/MS chromatogram doubles in size on day 2, it is likely that the air concentration also doubled on day 2. The assumptions would be that all of the same diffusion conditions were equal on both days and that errors in the relative quantitation would be related to a deviance in those assumptions (e.g., a doubling of wind speed).

Given that the activated carbon badge was deployed on the same day for exactly the same amount of time, it is logical to assume that both samplers encountered the same air volume from which to sample. While absolute quantitation cannot be assumed for the SPME data, a direct comparison of that integrated peak area on a compound-by-compound basis provides the relative difference in sensitivities between SPME and the activated carbon badges. Table 4 describes the differences in the integrated peak areas for the compounds detected by SPME, so relative sensitivities can be compared.

The data demonstrate that many of the compounds were present only on the SPME fiber. This suggests a greater applicability/versatility of the fiber from many of the VOCs and SVOCs measured. For this reason, our lab [43] and others [44,45] have previously used SPME to detect mold-related VOCs (MVOCs). The SPME fiber appears to generally be more sensitive as well. Twenty compounds were detected using SPME that were not seen with the activated carbon badge even though they have previously been observed [46], and only seven of the seventeen compounds detected by both techniques had a greater peak area with the carbon badge.

The airborne respirable dust samples were collected at the same time and on the same farmers that the VOC samples were collected. Thirty samples (one per farm) were collected during each farming activity. All samples collected during harvesting and 22 during land preparation had measurable levels of particulate. Conversely, most of the samples collected during pesticide application were lower than the detection limit of the microbalance. All samples were well below the NIOSH recommended value of 5 mg/m^3^ [47]. Table 5 presents the results of the respirable dust concentration to which farmers may have exposed themselves during farming. The highest concentrations measured occurred during harvesting, i.e., 1.83 mg/m^3^.

The symptoms related to chemical or particulate exposure were recalled from farmers’ experiences within 24 h farming activity (Table 6). Most farmers mentioned health effects related to their central nervous system and/or respiratory system. About half of the participants reported nausea (50.0%), and thirteen farmers experienced headaches (43.3%). The main respiratory symptoms were cough and runny nose (43.3%). For CNS symptoms, the participants indicated muscular twitching and cramps (26.7%). Few of them reported excessive salivation (6.7%), and abdominal pain or stomachache (13.3%). In total, 43% of respondents had eye irritation while 33.3% reported eye lacrimation and blurred vision. In addition, neuromuscular symptoms such as restlessness (30.0%), difficulty in seeing and falling asleep, irritability, and memory problems (20.0%) were also reported. However, only 5% of rice farmers reported trembling of their hands and anxiety after their exposure.

The correlation between symptoms and farmer’s work practice, farming activities factors and working environmental pollutants were analyzed using Pearson correlations and the Mann–Whitney U test. The results show that none of the work practice variables such as farm area, duration of working, and number of annual mechanical application per year were associated with health symptoms (*p* > 0.05) Likewise farming activities including land preparation, pesticide application, and harvesting had no association with health related symptoms (*p* > 0.05). Finally, working environmental pollutants, VOC concentration, and respirable dust were also not correlated with farmer-reported symptoms (*p* > 0.05).

## 4. Discussion

Safety instructions on containers are often written in unfamiliar languages. Since most of the participants only had an elementary school level of education, they might have difficulty reading or found instructions difficult to follow [48,49,50]. The respondents (86.7%) also indicated that they usually checked their equipment prior to use. This was similar to other studies performed in Thailand, where it was reported that about 97% of the farmers checked the condition of their equipment before using them [51]. Most respondents reported that they never smoked or drank alcohol while spraying pesticides, which confirmed the results of a similar study that reported more than half of their respondents not smoking or drinking water while working [52,53]. Most of the participants wore masks while spraying, which was relatively consistent with the results of other studies from Ethiopia and Thailand [49,54,55].

The results showed that most of the samples collected during pesticide application had detectable VOCs and SVOC compounds, in part because the farmers hung the mechanical sprayer on their shoulders, very close to their breathing zone. Since farmers may have been exposed to chemicals including VOCs, SVOCs, and pesticides, long-term exposure and the possible resultant health effects have been suggested for further study [54,55,56,57,58]. Eleven VOCs compounds were found in these air samples including Methlylene Chloride; Ethylbenzene; o-Xylene; m-Xylene; p-Xylene; Styrene; 1-Methyl Ethylbenzene; Tert-Butyl Benzene; 1, 3, 5-Trimethyl Benzene; 1-Methyl Propyle Benzene; and 1, 4-Dichloro Benzene. However, the farmers’ exposure to these chemicals were at less than 50% OELs. The agricultural machinery used primarily emits NOx, PM2.5, PM10, VOCs, HC, CO, and SO_2_ [10,59]. Our results were similar to those in previous studies measuring emissions produced by gasoline combustion engines used in agricultural machinery. For example, a similar study conducted in Korea reported that 15 types of VOC emissions from a gasoline engine were detected in or near the breathing zone of the applicator [60].

For respirable particulate exposure, the results showed that the highest concentration (1.83 mg/m^3^) occurred during harvesting. This was not unexpected as it is the dry process with high agitation. These results are similar to those in previous studies that reported dust generation from farming activities [22,23,24].

Most participants mentioned symptoms related to the central nervous system and the respiratory system. The average temperature in the study area was higher than 30 °C [61]. Previous studies reported that their symptoms increased in hot weather [62,63]. Thus, the symptoms that farmers thought were related to gasoline engine combustion by-products may actually come from working in hot weather. These reports were similar to other studies that found that central nervous system (CNS) symptoms resulted from farming-related “illnesses” while working in the field [64]. Moreover, a study in Iran reported that respiratory disorders are a common problem among farmers and that the prevalence rates of respiratory symptoms were higher than in non-farmers [65]. Some of the participants in this study also reported symptoms related to skin diseases, and neuromuscular symptoms, also observed in other studies that reported excessive sweating and dermal effects (skin rash/itching/burning), were common [66,67,68]. The neuromuscular problem reported in this study was the same as the most frequently reported symptom in a study in Brazil [67,68,69]. While the symptoms were similar to those from other studies, no significant associations between the reported symptoms and work practice, or environmental pollutants were found, possibly due to the small population. The lower levels of airborne contaminants measured and the breath of environmental insults (particles, heat, VOCs, etc.) may also make correlating symptoms more difficult. Therefore, the risk to farmers from multiple long-term low-level exposures to pollutants should continue to be of concern.

Both the singular crop rice and the limited study area, Pathumthani Province, could be considered limitations of the study. In addition, the fragility of SPME for air sampling needs to be reconsidered as most of the SPME samples were lost. The charcoal tube attached to a personal pump provided sufficient data as an alternative but adds weight and size that may interfere with the applicator movement and may also be less sensitive than SPME. Future studies could use either silicone wrist bands or SPME fabric as more rugged passive sampling monitors.

## 5. Conclusions

This study investigated VOCs, SVOCs, and respirable dust particulate in the air during rice farming, assessing for correlations with famer-reported symptoms related to the aforementioned chemical exposure. While limited, the contaminant data showed that, in addition to pesticides, rice farmers may be exposed to other pollutions such as VOCs and SVOCs as combustion byproducts (e.g., Toluene and Xylene) from farming activities that use gasoline-powered equipment. A comparison of air samplers showed SPME to be more sensitive than the activated carbon badge but much more fragile as well. CNS and dermal irritation symptoms reported by participants in this study were like those from other studies using similar farming practices and equipment.

The data demonstrated that VOC and SVOC exposure most often occurred during land preparation and pesticide application and that the highest dust particle exposure occurred during harvesting, which may lead to many respiratory health effects or at least farmer-reported symptoms. This cross-sectional study focused on rice farmers and may provide baseline data for relevant regulatory agencies. As a follow-up, policy implementation and risk communication will be introduced to the community to develop safety programs and to sustain improvement in behaviors. The implementation of public health education training programs for rice farmers to improve their working conditions and quality of life, including providing mitigation strategies for chemical exposure and information on appropriate personal protective equipment (PPE), is also suggested. Future studies should include additional chemical contaminants, measurement devices, and bio-monitoring of participants.

## Figures and Tables

**Table 1:** Sociodemographic and occupational experience of the respondents (*n* = 30).

Characteristics	Frequency	Percentage
Age (years old)		
<30	2	6.7
31–40	3	10.0
41–50	10	33.3
51–60	9	30.0
>60	6	20.0
Min = 23 Max = 78		
Mean + SD: 50 + 12.7		
Education		
None	1	3.3
Primary School	17	56.7
Secondary School	6	20.0
High School	5	16.7
Bachelor’s Degree and above	1	3.3
Family income (Baht/Year)		
<50,000	5	16.7
50,001–100,000	14	46.6
100,000–150,000	6	20.0
>150,000	5	16.7
Smoking		
Never	6	20.0
Used to smoke	7	23.3
Currently smoking	17	56.7
Congenital or underlying disease		
Yes	13	43.3
No	17	56.7
Currently on any medication		
Yes	9	30.0
No	21	70.0
Agriculture experiences (Year(s))		
<10	11	36.7
11–20	7	23.3
21–30	6	20.0
>30	6	20.0
Years using mechanical applications (Year(s))		
Tractor		
<10	12	40.0
11–20	8	26.7
21–30	6	20.0
>30	4	13.3
Mechanical knapsack sprayer		
<10	12	40.0
11–20	9	30.0
21–30	5	16.7
>30	4	13.3

**Table 2:** Farm and work practices (*n* = 30).

Characteristics	Frequency	Percentage
Farm area (rai(s))		
<10	17	56.7
11–20	6	20.0
21–30	2	6.6
>30	5	16.7
(1 rai = 1600 square meters)		
Duration of working (h/time)		
Land preparation		
<1	-	-
1–2	14	46.7
>2	16	53.3
Pesticide spraying		
<1	30	100.0
1–2	-	-
>2	-	-
Harvest		
<1	-	-
1–2	26	86.7
>2	4	13.3
Number of annual mechanical applications (time(s)/Year)		
Tractor		
<10	13	43.3
11–20	13	43.3
21–30	3	10.0
>30	1	3.3
Mechanical backpack sprayer		
<10	7	23.3
11–20	12	40.0
21–30	2	6.6
>30	9	30.0
Handling and work practice		
Follows all product instructions	27	90.0
Checks tools before use	30	100.0
Prepares mechanical applications at home	26	86.7
Stores mechanical applicators at home	26	86.7
Cleans spraying equipment after work	29	96.7
Takes a meal at work place	12	40.0
Smokes while applying pesticides	6	20.0
Considers the safety period	28	93.3
PPE usage		
Gloves		
Rubber	26	86.7
Fabric	26	86.7
Long	26	86.7
Short	26	86.7
Mask	26	86.7
Boots	24	80.0
Hat	29	96.7
Short sleeved shirt	5	16.7
Long sleeved shirt	29	96.7
Short sleeved pants	2	6.6
Long sleeved Pants	28	93.3

**Table 3:** Detection frequency and average concentrations of VOCs in personal samples.

Activities	Chemicals	Concentration	OEL
Land preparation (*n* = 30)	Methlylene Chloride	≤10% at OEL	PEL = 25 ppm [34]
	1, 1, 1 Trichloro Ethane		TWA = 350 ppm ^a^
	Benzene		TWA = 1 ppm [35]
	Ethylbenzene		TWA = 100 ppm ^a^
	o-Xylene		TWA = 100 ppm ^a^
	m-Xylene		TWA = 100 ppm ^a^
	p-Xylene		TWA = 100 ppm ^a^
	Styrene		TWA = 50 ppm ^a^
	1-Methyl Ethyl Benzene		TWA = 50 ppm [36]
	1, 3, 5-Trimethyl Benzene		TWA = 25 ppm [37]
	1, 4-Dichloro Benzene		TWA = 75 ppm ^a^
	Naphthalene	≤50% at OEL	TWA = 10 ppm [38]
	Tert-Butyl Benzene	No occupational exposure limits ^a^	
	1-Methyl Propyle Benzene		
	1, 2, 3 Trichloro Benzene		
Pesticide application (*n* = 30)	Methlylene Chloride	≤10% at OEL	PEL = 25 ppm [34]
	1, 1, 1 Trichloro Ethane		TWA = 350 ppm ^a^
	Ethylbenzene		TWA = 100 ppm ^a^
	o-Xylene		TWA = 100 ppm ^a^
	m-Xylene		TWA = 100 ppm ^a^
	p-Xylene		TWA = 100 ppm ^a^
	Styrene		TWA = 50 ppm ^a^
	1-Methyl Ethyl Benzene		TWA = 50 ppm [36]
	1, 3, 5-Trimethyl Benzene		TWA = 25 ppm [37]
	Naphthalene	≤50% at OEL	TWA = 10 ppm [38]
	Tert-Buty1 Benzene	No occupational exposure limits ^a^	
	1-Methyl Propyle Benzene		
	1, 2, 3 Trichloro Benzene		
Harvesting (*n* = 30)	1, 1, 1 Trichloro Ethane	≤10% at OEL	TWA = 350 ppm ^a^
	Ethylbenzene		TWA = 100 ppm ^a^
	o-Xylene		TWA = 100 ppm ^a^
	m-Xylene		TWA = 100 ppm ^a^
	p-Xylene		TWA = 100 ppm ^a^
	1-Methyl Ethyl Benzene		TWA = 50 ppm [36]
	1, 3, 5-Trimethyl Benzene		TWA = 25 ppm [37]
	Naphthalene	≤50% at OEL	TWA = 10 ppm [38]
	Tert-Butyl Benzene	No occupational exposure limits ^a^	
	1-Methyl Propyle Benzene		
	1, 2, 3 Trichloro Benzene		

Note: OEL: occupational exposure limit, TWA: time-weighted average, PEL: permissible exposure limit, ^a^ = The National Institute for Occupational Safety and Health (NIOSH), 2018 [39].

**Table 4:** The comparison between the compounds detected by SPME and charcoal tube.

SPME Samples			
Activities	Chemicals	Charcoal Tube Found	Comparison to Charcoal Tube Concentration
Pesticide application (*n* = 3)	1, 2 Dichloro Ethaene	ND	
	Methlylene Chloride	Detected	Higher
	Trichloro Methane	ND	
	Toluene	ND	
	Dibromo Chloro Methane	ND	
	1, 2 Dibromo Ethane	ND	
	Ethylbenzene	Detected	Higher
	o-Xylene	Detected	Lower
	m-Xylene	Detected	Lower
	p-Xylene	Detected	Higher
	Styrene	Detected	Higher
	1-Methyl Ethyl Benzene	Detected	Higher
	1-Chloro-2-Methy-lBenzene	ND	
	1, 3, 5-Trimethyl Benzene	Detected	Higher
	1, 3-Dichloro Benzene	ND	
	1, 4-Dichloro Benzene	Detected	Lower
	1, 3, 5 Trichloro Benzene	ND	
	1, 3 Dichloro Propane	ND	
	1, 2 Dibromo-3-Chloro Propane	ND	
	Propyl Benzene	ND	
	1-Chloro-3-Methyl-Benzene	ND	
	1-Ethyl-3-Methyl Benzene	ND	
	Tert-Butyl Benzene	Detected	Lower
	1-Methyl Propyle Benzene	Detected	Lower
	Butyl Benzene	ND	
Harvesting (*n* = 1)	Methlylene Chloride	ND	
	1, 2 Dichloro Propane	ND	
	Toluene	ND	
	Ethylbenzene	Detected	Higher
	o-Xylene	Detected	Lower
	m-Xylene	Detected	Lower
	p-Xylene	Detected	Higher
	Stylene	Detected	Higher
	1, 3, 5-Trimethyl Benzene	Detected	Higher
	1, 3 Dichloro Propane	ND	
	Propyl Benzene	ND	
	1-Chloro1-3-Methyl-Benzene	ND	

Note: ND = not detected.

**Table 5:** Summary of personal respirable dust concentrations (mg/m^3^).

Farming Activities	No. of Sample	Percent Measurable	Avg. Respirable Dust Conc. (Range) (mg/m^3^)
Land preparation	30	73.3%	0.03 (0.00–0.34)
Pesticide application	30	13.3%	0.00 (0.00–0.09)
Harvesting	30	100%	0.49 (0.04–1.83)

**Table 6:** Symptoms related to chemicals exposure (*n* = 30).

Symptoms	Frequency	Percentage
Skin Symptoms		
Skin rash/itching/burning	6	20.0
Tingling/numbness of hands	9	30.0
muscular twitching and cramps	8	26.7
Respiratory Symptoms		
Chest pain	4	13.3
Cough	13	43.3
Running nose	13	43.3
Difficulties in breathing	12	40.0
Shortness of breath	7	23.3
Irritation of the throat	12	40.0
Central Nervous System Symptoms		
Excessive sweating	9	30.0
Nausea	15	50.0
Vomiting/Dizziness	6	20.0
Excessive salivation	2	6.7
Abdominal pain/Stomachache	4	13.3
Headache	13	43.3
Eye Symptoms		
Lacrimation	10	33.3
Irritation	13	43.3
Blurred Vision	10	33.3
Neuro Muscular Symptoms		
Difficulty in seeing	6	20.0
Restlessness	9	30.0
Difficulty in failing asleep	6	20.0
Trembling of hands	5	16.7
Irritability	6	20.0
Anxiety/anxiousness	5	16.7
Memory Problems	6	20.0
